# Pooling multimodal cancer data across unaligned embedding spaces maintains tumor of origin signal

**DOI:** 10.1093/bioadv/vbag159

**Published:** 2026-07-29

**Authors:** Raphael Kirchgaessner, Kaya Keutler, Shruthilayaa Sivakumar, Xubo Song, Kyle Ellrott

**Affiliations:** BioMedical Engineering, Oregon Health and Science University, Portland, OR 97201, United States; BioMedical Engineering, Oregon Health and Science University, Portland, OR 97201, United States; Center for Biomedical Data Science (CBDS), Knight Cancer Research Institute, Portland, OR 97201, United States; Center for Biomedical Data Science (CBDS), Knight Cancer Research Institute, Portland, OR 97201, United States; Division of oncological sciences, Knight Cancer Research Institute, Portland, OR 97201, United States; BioMedical Engineering, Oregon Health and Science University, Portland, OR 97201, United States; Center for Biomedical Data Science (CBDS), Knight Cancer Research Institute, Portland, OR 97201, United States

## Abstract

**Summary:**

AI-based embeddings offer the possibilities of encoding complex biological data into low-dimensional spaces, called embedding spaces, that maintain the relationships between entities. Vector pooling is the process of aggregating an array of embedded vectors, either usually by summing or averaging, to summarize the total movement with the embedding space. Embedded vector pooling allows sampling of an arbitrary number of points to be summarized into a fixed sized vector, and is frequently used to sample networks of embedded values or to summarize protein language model vectors. There is an open question about the compatibility of embedding spaces that are created without any coordination. It has been assumed that signals in these unaligned embedding spaces would be destroyed if vectors were pooled into summed values. To challenge this idea, we created a number of benchmarks that utilized unaligned embedded values and pooled them into heterogeneous vectors to test information retrieval. To power this benchmark, we trained embedding models across different cancer data modalities and tested how well pooled heterogeneous vectors were able to retain biologically relevant information. Our research shows that signal from unaligned embedded values is conserved and able to still be used for learning tasks, such as data modality and tumor of origin recognition.

**Availability and implementation:**

All code and computational experiments related to this publication can be found at https://github.com/EllrottLab/heterogeneous-embedding-vectors.

## 1 Introduction

As AI techniques are applied to biomedical and genomic data, questions arise regarding data encoding and representation. Most modern AI pipelines begin by projecting raw source data into a latent embedding space (Gema *et al.* 2024, Madan *et al.* 2024, Waqas *et al.* 2024). In these lower-dimensional manifolds, relative relationships are maintained such that semantically similar elements are positioned closer together. In fields such as natural language processing and computer vision, combining diverse data into single vectors via pooling functions, specifically summation, is common practice (Chen *et al.* 2021, Naderializadeh *et al.* 2021, Naderializadeh and Singh 2025). However, such pooling is typically reserved for homogeneous data (e.g. combining word embeddings in a sentence). In biological settings, while single modalities like proteomics are treated as homogeneous, a holistic clinical perspective requires integrating heterogeneous sources, such as transcriptomics, pathology images, and clinical notes, to accurately represent disease complexity (Amal *et al.* 2022, Wang *et al.* 2025).

Traditional approaches to multi-omic integration often rely on joint modeling or coordinated optimization frameworks, such as the Contrastive Language-Image Pre-Training (CLIP) algorithm or linear alignment methods (Cantini *et al.* 2021, Radford *et al.* 2021). While effective, these methods require pre-planned optimization and are computationally expensive to scale as new modalities are added. In contrast, simple sum-pooling is a near-instantaneous operation with O(1) time complexity. This allows it to be integrated “on-the-fly” within larger algorithmic frameworks without the need for a full joint-alignment retraining cycle.

A central concern raised by the use of unaligned pooling is the risk of “interference.” In coordinated spaces, axes are optimized to prevent overlap of distinct concepts. In unaligned spaces, there is no such guarantee. However, we argue that the emergence of foundation models in biology changes this calculus. Unlike early variational autoencoders (VAEs) that sought the minimum number of dimensions (often 50–100) to model a single phenotype (Way and Greene 2018, Chow *et al.* 2022), modern foundation models utilize significantly larger latent spaces to ensure the model is useful across a broad spectrum of downstream tasks.

For instance, transcriptomic foundation models like GeneFormer (Zheng and Gao 2023) utilize 768 to 1152 dimensions, while protein models such as Evolutionary Scale Modeling (ESM) Yang *et al.* (2026) range from 320 up to 5120 dimensions. Similarly, pathology image models like GigaPath (1536 dimensions) (Xu et al. 2024) and Midnight 12K (Karasikov *et al.* 2025) (3072 dimensions) provide high-capacity representations (TRIDENT) (Vaidya *et al.* 2025, Zhang *et al.* 2025). We hypothesize that these high-dimensional spaces are sparse enough that information loss during unaligned pooling is minimal, as the “signal” from each modality is likely to occupy distinct, non-conflicting manifolds within the broad latent space.

In this study, we investigate whether a simplistic strategy for multimodal integration, pooling unaligned, heterogeneous embeddings, can maintain the integrity of “Tumor of Origin” signals for search, clustering, and recognition. Our motivation is to determine if patient data (RNA transcription, H&E images, gene mutations and clinical text) can be fused into a unified vector without coordinating the manifolds beforehand. If the signal survives this pooling, it suggests that unaligned pooling can serve as a highly efficient, modular component in complex graph-learning algorithms and vector database retrieval systems, facilitating the rapid development of personalized medicine tools.

## 2 Approach

**Figure 1:**
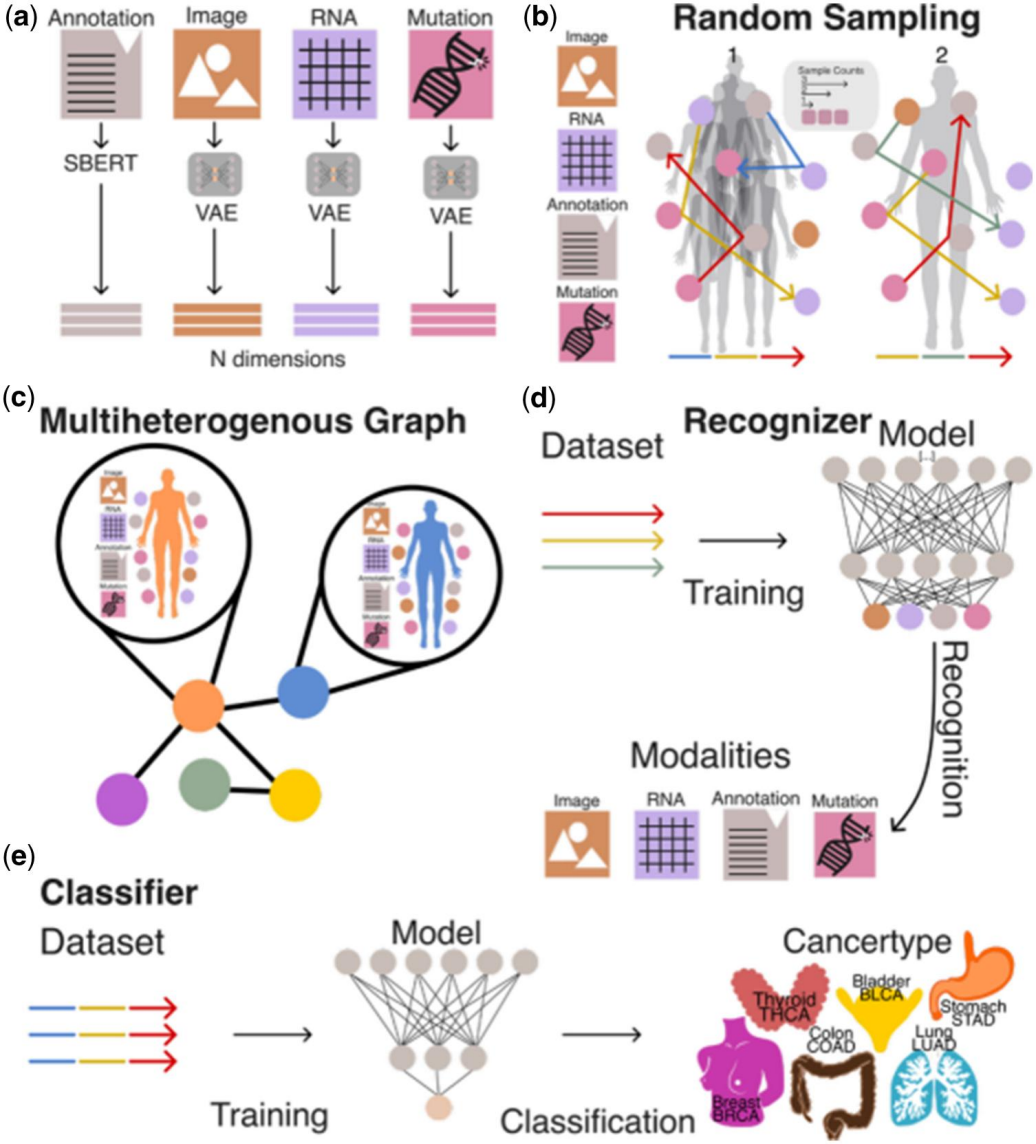
Overview of embedding generation and network architecture. (a) Modalities (RNA, Image, Mutation, and Annotation) used to generate embeddings by using either SBERT, vision transformer or a VAE. (b) Illustration of random samplings performed over multiple patients (1) and one patient (2), demonstrating different sampling counts. (c) Schematic representation of a heterogeneous graph incorporating patients and their associated modalities. (d) Schematic of the recognizer experimental setup to recognize multiple modalities, including image, RNA, annotations, and mutation data. (e) Schematic of the classifier experiment setup which predicts the patient’s cancer type, classified as BRCA, LUAD, BLCA, THCA, COAD, or STAD.

To investigate whether simple pooling of embeddings from unaligned latent spaces can yield meaningful representations for downstream tasks, we developed two complementary sampling and evaluation strategies. The first strategy focused on modality-level sampling to address the composition recognition problem. In this setting, the goal was to determine whether it is possible to infer which data modalities (e.g. RNA, H&E, clinical annotations) were used to construct a given pooled vector. To generate the training data for this task, we randomly sampled embeddings from different modalities without regard to patient identity, and combined them to form pooled vectors. A recognition model was then trained to predict the constituent modalities present in each input vector. This approach allowed us to assess whether naive pooling preserves sufficient modality-specific structure to support decomposition, despite the embeddings originating from unaligned latent spaces. The second strategy was designed to assess the clinical and biological utility of the pooled embeddings. Here, the task was to predict cancer-relevant attributes—including cancer type [e.g. breast cancer (BRCA), bladder cancer BLCA], tumor mutational burden (TMB), and molecular subtype—based on patient-level pooled vectors. To construct these vectors, we performed patient-level sampling, where each pooled vector was composed of heterogeneous embeddings corresponding to the same individual. These included representations derived from RNA-seq profiles, H&E images, clinical annotations, and somatic mutation data. This setup enabled us to evaluate whether the pooled vectors retained enough biological signal to be useful for clinically relevant prediction tasks. All embeddings were generated using either VAEs or Sentence-BERT (sBERT) (Fig. 1a). Each embedding represented a datapoint tied to a specific patient or modality. The only coordination between different embedding models was dimensionality alignment: all vectors were projected into a shared 768-dimensional space to match the output size of the sBERT model. To prepare the embeddings for use with traditional machine learning pipelines and vector databases, we applied structured random sampling and pooling procedures. This transformation was critical for enabling efficient storage, retrieval, and analysis of heterogeneous data types using conventional vector-based methods. By encapsulating diverse multimodal representations into a unified vector format (Fig. 1b and c), we facilitated systematic evaluation of whether naive pooling preserves informative structure across both tasks. Together, these two strategies allowed us to evaluate the extent to which simple vector pooling from unaligned modality-specific embeddings can support downstream machine learning applications, including modality composition recognition and clinically meaningful phenotype prediction (Fig. 1d and e).

## 3 Methods

### 3.1 Creation of dataset

To obtain the necessary data, we utilized The Cancer Genome Atlas (TCGA), a comprehensive repository containing molecular and clinical data from over 10 000 cancer patients across 33 cancer types. For this study, we focused on six cancers: breast cancer (BRCA), bladder cancer (BLCA), lung adenocarcinoma (LUAD), stomach adenocarcinoma (STAD), thyroid cancer (THCA), and colon adenocarcinoma (COAD). TCGA provides extensive genomic insights, including RNA expression profiles, somatic mutations, and imaging data such as H&E-stained slides, enabling a robust analysis of cancer heterogeneity and multi modal embedding poolings.

### 3.2 Data preparation

For each cancer type, we gathered the corresponding H&E images, patient annotations, gene mutations, and RNA readouts. Specifically, we obtained data from patients diagnosed with either BRCA, BLCA, LUAD, THCA, COAD, or STAD cancer across these six cancer types (Tables 1 and 2). While image, RNA, and mutation data were readily available in formats like CSV and TIFF files, patient annotations were only provided in PDF format, making them less accessible for analysis. To facilitate processing, we converted the PDF files into text. For cases where the PDFs contained handwritten notes, we used Optical Character Recognition (OCR) to extract the textual information. The extracted text was then segmented by sentences or periods, producing multiple distinct text segments for each patient. Each segment was then transformed into separate embeddings, allowing us to create a comprehensive set of embeddings for every individual patient. This multi-layered embedding strategy ensured that all relevant data modalities were captured and integrated accurately for further analysis.

**Table 1:** Number of patients available per data modality.^a^

Modality	Patients
RNA	3558
Mutation	3189
H&E	3442
Annotations	3443

a RNA data includes the largest cohort with 3558 patients, while mutation data includes the smallest cohort with 3189 patients.

**Table 2:** Detailed distribution of available embeddings per cancer type across data modalities.^a^

Modality	BRCA	BLCA	COAD	STAD	THCA	LUAD
RNA	1000	430	514	453	572	589
Mutation	1003	407	404	431	435	509
H&E	329 400	123 600	138 000	132 900	152 100	156 600
Annotations	69 366	40 236	14 980	12 595	30 136	37 452

a Each modality includes at least one embedding per patient. RNA and mutation modalities consistently provide one embedding per patient. In contrast, annotations and H&E image modalities often include multiple embeddings per patient, with the image modality exhibiting the widest distribution.

### 3.3 Embedding generation

To capture the unique characteristics of each modality—RNA expression, somatic mutations, histopathology (H&E) images, and patient-level annotations—we generated modality-specific embeddings designed to reside in a shared representational space. Given the inherent heterogeneity in feature types and dimensionalities across these data sources, our objective was to project each modality into a compatible latent space that retained biological relevance while enabling downstream integration.

For RNA, mutation, and image data, we employed VAEs to learn compact, biologically meaningful latent representations. For patient-level annotations, we leveraged a pretrained sBERT model, which produces 768-dimensional embeddings optimized for semantic similarity in textual data (Reimers and Gurevych 2019). To align all modality representations, each VAE was configured to output embeddings of 768 dimensions, matching the fixed dimensionality of sBERT. This uniform representation allowed for straightforward pooling and cross-modality comparison.

Each VAE was trained separately on data from all six cancer types to ensure generalizability and to preserve the biological variability inherent across different tumor types. After training, embeddings were extracted using the encoder component of each VAE, enabling compression of modality-specific input into a biologically informative latent space.

For somatic mutations, the mutation VAE was trained on a one-hot encoded mutation matrix. Training was performed with a batch size of 256 and incorporated an early stopping criterion with a patience of 10 epochs to prevent overfitting. A maximum of 50 training epochs was allowed. The resulting encoder produced a 768-dimensional latent vector that effectively captured mutational patterns across patients. For RNA expression data, a dedicated VAE was trained using RNA-seq data from TCGA. Training was performed in two stages: an initial pretraining phase followed by fine-tuning. A warm-up strategy was applied to the Kullback-Leibler (KL) divergence term in the VAE loss function to improve training stability. Specifically, KL loss was initialized at zero and gradually increased according to an annealing schedule governed by a κ (kappa) parameter. In our implementation, κ was set to 1, and the KL loss weight (β) was initialized at 0, resulting in a one-epoch warm-up phase. This annealing technique is known to promote more stable convergence by allowing the model to learn meaningful reconstructions before imposing latent space regularization.

For H&E histopathology images, whole-slide images were partitioned into non-overlapping 256 × 256 pixel tiles. Tiles predominantly containing background were removed using a simple RGB pixel thresholding heuristic. Remaining tiles were processed with Prov-Gigapath, a whole-slide foundation model pre-trained on large-scale pathology datasets (Xu *et al.* 2024). Tile-level inference produced 1536-dimensional embeddings, which were then truncated to the final 768 dimensions. This truncation strategy was informed by internal benchmarking, in which we compared pairwise distances in PCA space between the original 1536-dimensional embeddings and their truncated 768-dimensional counterparts, confirming that the truncation preserved the geometric structure of the representation and retained sufficient discriminative signal for downstream tasks.

### 3.4 Embedding pooling

To construct a comprehensive dataset for training and evaluating recognition models, we generated pooled embeddings composed of 3 to 10 constituent embeddings per vector. This range ensured exposure to varying levels of pooling complexity, enabling the model to generalize across different embedding combinations. The embedding construction process followed two distinct sampling strategies: the simple pooling approach and the specific-cancer approach, corresponding to our previously described first and second strategies, respectively. For the simple pooling approach, all available embeddings across modalities—RNA, H&E, mutations, and annotations—were first loaded into memory. A random sampling procedure was applied, selecting a specified number of embeddings (e.g. sample count of 3 to 10) regardless of their patient or cancer type of origin. The selected embeddings were summed to produce a new 768-dimensional pooled embedding, and the modalities contributing to the pooling were tracked and stored as ground truth labels for supervised training. In the specific-cancer approach, the sampling procedure was constrained to enforce cancer-type specificity. After loading all available embeddings, random sampling was performed such that all selected embeddings originated from the same cancer type (e.g. only embeddings derived from BRCA patients). As in the simple approach, the selected embeddings were summed to form a 768-dimensional vector. Both the modalities involved in the pooling and the corresponding cancer type were recorded as ground truth labels. This design enabled us to evaluate the recognizer model’s performance under both general and cancer-specific conditions, while preserving control over the composition and structure of the pooled embeddings.

#### 3.4.1 Training procedure

The recognizer network was trained using supervised learning. The input to the model was the pooled heterogeneous embedding vector, and the output was a set of per-modality count predictions indicating how many embeddings of each modality contributed to the pool, framed as a multi-class classification problem with classes ranging from 0 to the maximum walk distance. The model was trained using the Adam optimizer with an initial learning rate of 0.001, and categorical cross-entropy loss was applied independently to each modality output. The network architecture consisted of an initial set of shared layers designed for general feature extraction (512→256→128→64 units), followed by modality-specific output branches. Each branch is projected directly from the shared representation to the output space via a softmax layer. To improve generalization and stability, the architecture incorporated fully connected layers, Dropout (rate = 0.3), and batch normalization after each layer of the shared backbone. Training proceeded in two phases: an initial joint optimization of all parameters, followed by a fine-tuning phase in which the shared backbone was frozen and task-specific branches were further refined at a reduced learning rate. Early stopping was applied in both phases to prevent overfitting. To prevent modality imbalance during training, the probability of selecting each modality during the random sampling process was explicitly controlled. When using all four modalities, each was assigned a uniform selection probability of 25%, ensuring equal representation across training batches and avoiding bias toward more frequently represented modalities.

#### 3.4.2 Validation and testing

The dataset was split into train, and test sets using an 80/20 split, followed by an 80/20 subdivision of the training set into training and validation subsets. The validation set was used during training to monitor model generalization and tune hyperparameters, while the test set was held out entirely for final performance evaluation.

#### 3.4.3 Composite recognizer model

In addition to training individual isolation models for each sample count, we trained a composite recognizer model using a merged dataset containing all pooled embeddings with sample counts from 3 to 10. This model was designed to assess whether a single network could generalize across varying levels of pooling complexity and modality combinations. The same training architecture and optimization parameters were applied to the composite model, enabling direct comparison with the isolation-based models.

#### 3.4.4 Metric calculation for recognizer network evaluation

Evaluating the recognizer network required special consideration because the training and test datasets generated under the modality-level sampling strategy were highly sparse. In this sampling framework, embeddings were randomly selected across modalities—without regard to patient identity—and pooled to form synthetic vectors containing between 3 and 10 constituent embeddings. As any given modality could be present or absent in a pooled vector, the resulting binary label matrix naturally contained a high proportion of zeros. Additional constraints in the data-generation pipeline (e.g. limiting available embeddings or excluding particular modalities) introduced further systematic zeros in specific label columns.

This sparsity poses challenges for traditional metrics such as accuracy, mean absolute error (MAE), and mean squared error (MSE), all of which can be artificially inflated by the large number of correctly predicted zeros. For example, a trivial model that predicts “absent” for all modalities would obtain deceptively high accuracy despite having no ability to detect true modality presence. To avoid these pitfalls, we evaluated model performance primarily using the F1 score, balanced accuracy, and the Matthews correlation coefficient (MCC). These metrics are well-suited for imbalanced data: F1 focuses exclusively on the model’s ability to recover present modalities, and MCC provides a symmetric, correlation-based measure that penalizes models that fail to identify positives, even when true negatives are abundant. Importantly, these metrics are already robust to zero inflation and do not become inflated by large numbers of correct zero predictions, making it unnecessary to compute separate F1 or MCC values for zero and non-zero subsets.

We additionally evaluated model robustness under controlled perturbation. In this setting, Gaussian noise replaced a subset of constituent embeddings within each pooled vector while keeping the total sample count fixed. Because noise substitution removes true modality contributions, the corresponding ground-truth labels become even sparser. Under these conditions, raw accuracy again fails to distinguish between genuine compositional reasoning and trivial all-zero predictions. F1, balanced accuracy, and MCC remain informative in this setting because they directly quantify the model’s ability to identify true positive modality contributions even when noise obscures substantial portions of the pooled vector. Together, these evaluation choices ensured that performance metrics reflected the recognizer network’s true ability to infer the constituent modality composition of pooled embeddings—both in clean conditions and under structured noise—while avoiding misleading inflation caused by zero-dominant label structure.

### 3.5 Gaussian mixture models

Gaussian mixture models (GMMs) were used to evaluate the intrinsic structure of the pooled embedding space and to assess the extent to which cancer-type identity is recoverable without supervised learning. GMMs represent the data as a probabilistic mixture of multivariate Gaussian components and infer component parameters via expectation-maximization, allowing flexible modeling of non-spherical and partially overlapping clusters. Models were fit with a predefined number of components corresponding to the number of cancer types, or, when specified, by selecting the optimal number of components and covariance structure using Bayesian Information Criterion minimization. Cluster assignments were obtained based on maximum posterior probability.

To quantify clustering quality and latent separability, we computed silhouette scores using both Euclidean and cosine distances. Silhouettes were calculated with respect to the true cancer labels as well as the GMM-derived cluster labels, providing complementary measures of intrinsic cancer-type cohesion and unsupervised cluster compactness. For each cancer type, intra-class and nearest inter-class mean distances were computed, and class-level silhouette estimates were derived analytically from these distances to characterize local separability.

Concordance between GMM clusters and cancer labels was assessed using several complementary alignment metrics. Global correspondence was quantified using the adjusted rand index, normalized mutual information, and homogeneity-completeness-V measures. From the contingency table relating cancer types to GMM components, we computed cluster purity and class coverage to evaluate dominance patterns and label concentration. Optimal one-to-one cluster-label assignments were obtained using the Hungarian algorithm, from which we reported assignment accuracy and macro-averaged F1 scores.

A global chi-square test was used to assess the overall statistical dependence between cancer labels and cluster assignments. All metrics and summary statistics were retained for downstream interpretation and visualization.

### 3.6 Classification model

The classification model was trained using a supervised learning approach, with input features consisting of pooled and concatenated patient-level embeddings constructed via the second sampling strategy (patient-level sampling). Each embedding was paired with a ground truth label corresponding to one of three classification targets: cancer type, cancer subtype, or TMB.

Input normalization was performed in-network via batch normalization applied as the first operation of the model, prior to any learned transformations. Rather than applying fixed preprocessing transformations, batch normalization was used to adaptively normalize the input distribution during training, as it has been shown to smooth the optimization landscape and stabilize gradient flow without relying on precomputed dataset statistics (Ghahremani and Wachinger 2023). To account for class imbalance across cancer types, class weights were applied during training, up-weighting underrepresented classes to prevent the model from biasing toward majority classes.

Data were split using an 80/20 train-test split, with training and testing set ratios held consistent across all experimental runs. To ensure reliable and robust performance estimation, patients were solely assigned to either the train set or the test set. The train set was then split further into a train and validation set and the model was trained and evaluated across at least 30 independent runs using the three different datasets. This repeated training procedure reduced the impact of stochastic variability introduced by random initialization and data shuffling, and enabled the calculation of stable performance metrics across runs. Results were pooled to report average performance, allowing for a more accurate and reproducible assessment of the model’s predictive capacity.

### 3.7 TMB calculation

To calculate the TMB for each patient, we used the one-hot encoded somatic mutation file, which was used to generate the mutation embeddings, where each gene’s mutation status was encoded as either mutated (1) or not mutated (0). For each patient, we counted the number of mutated genes by summing all values across the remaining columns.

To standardize the calculation, we assumed an exonic coverage of 30 megabases (Mb) and computed TMB as the number of mutations per megabase using this formula: TMB = number of mutations/30 (Zhou et al. 2021). We then classified TMB into high and low categories based on a predefined threshold. A TMB value of 0.5 mutations/Mb or higher was classified as high (1), while values below this threshold were classified as low (0). If no mutations were detected for a patient, TMB was set to 0, and the classification was assigned a separate category (2) to account for cases where no mutations were present.

This approach ensures a consistent and interpretable quantification of TMB across patients while enabling classification into biologically relevant groups.

### 3.8 Tumor subtypes

We utilized publicly available resources to map patients and cancer types to their corresponding cancer subtypes. Specifically, subtype annotations were derived using data and tools from the reference file from the article “Classification of non-TCGA cancer samples to TCGA molecular subtypes using compact feature sets” (Ellrott *et al.* 2025) available through the Genomic Data Commons (GDC) and the subtype mapping file provided in the associated repository [https://github.com/NCICCGPO/gdan-tmp-models]. Subtype classification was performed only for cancer types where a sufficient number of annotated samples (n>100) were available to support reliable supervised learning.

### 3.9 Statistical analysis

Statistical comparisons between independent groups were performed using the two-sided Mann-Whitney *U* test, a non-parametric approach chosen to avoid assumptions of normality. To account for multiple comparisons, *P* value were adjusted using the Benjamini-Hochberg (BH) procedure. All statistical analyses and annotations were implemented via the “statannotations” library in Python. Significance levels are reported in figures as follows:

**** (P<.0001)

*** (P<.001)

** (P<.01)

*        P≥.05

*ns*       (not significant, P≥.05)

## 4 Results

### 4.1 Unsupervised clustering

**Figure 2:**
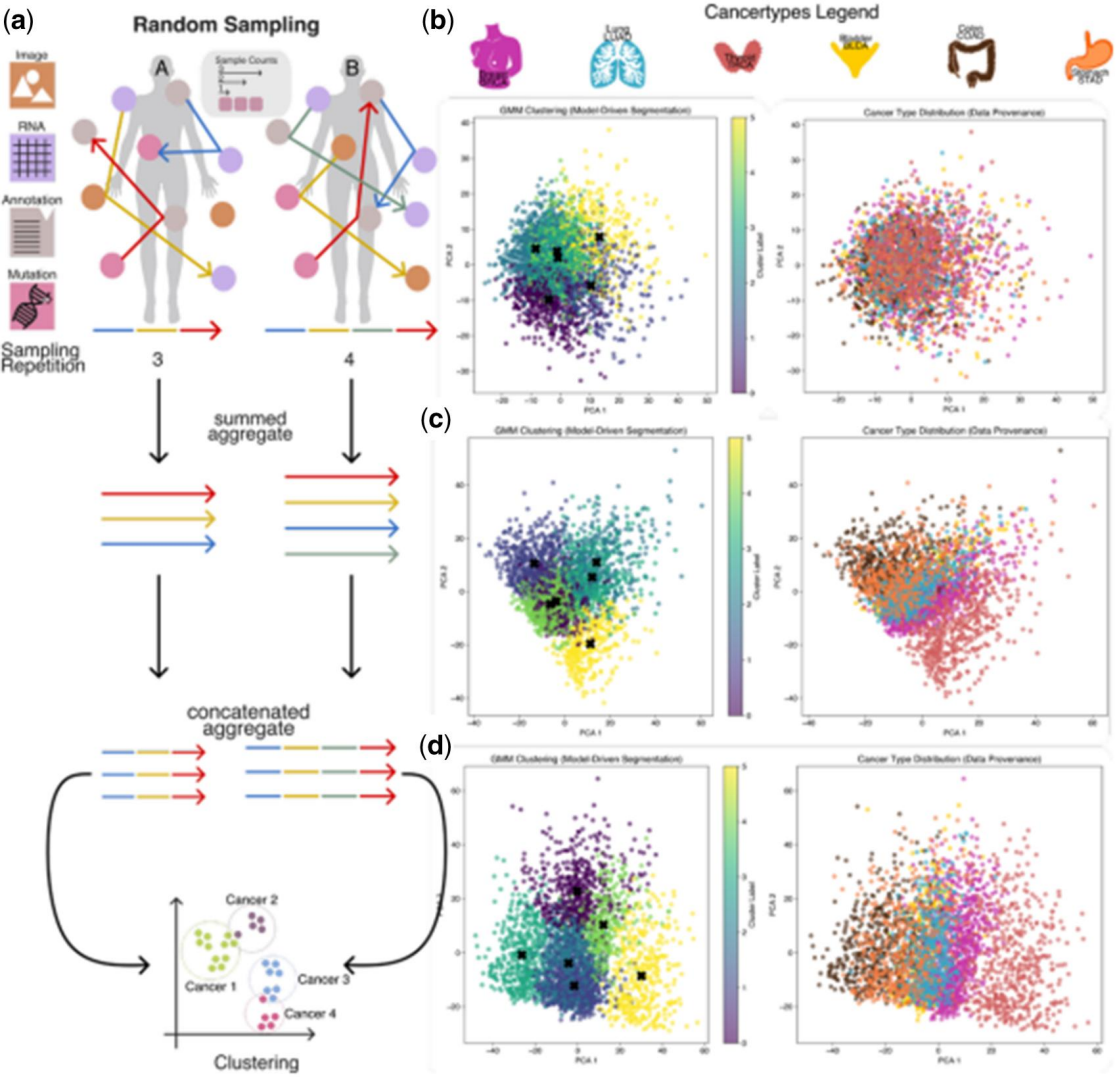
Unsupervised clustering of summed and concatenated embeddings. (a) Schematic depiction of patient-specific embeddings obtained by pooling and concatenating vectors derived from random samplings. Random samplings performed on the graph structure generate vectors concatenated into a single embedding vector for subsequent usage in a gaussian mixture model (GMM) clustering. (b) PCA representation of pooled and contacted embeddings using a sample count of 3 and a sample count repeat of 3, shows little differentiation between cancer types. (c) Increasing both sample count and repetition by 1 to 4 both PCA and GMM clustering shows a clearer separation between cancer types. (d) Using a sample count and repeat of 5 further enhances cancer type separation with THCA (far right cluster) observing the strongest separation.

**Figure 3:**
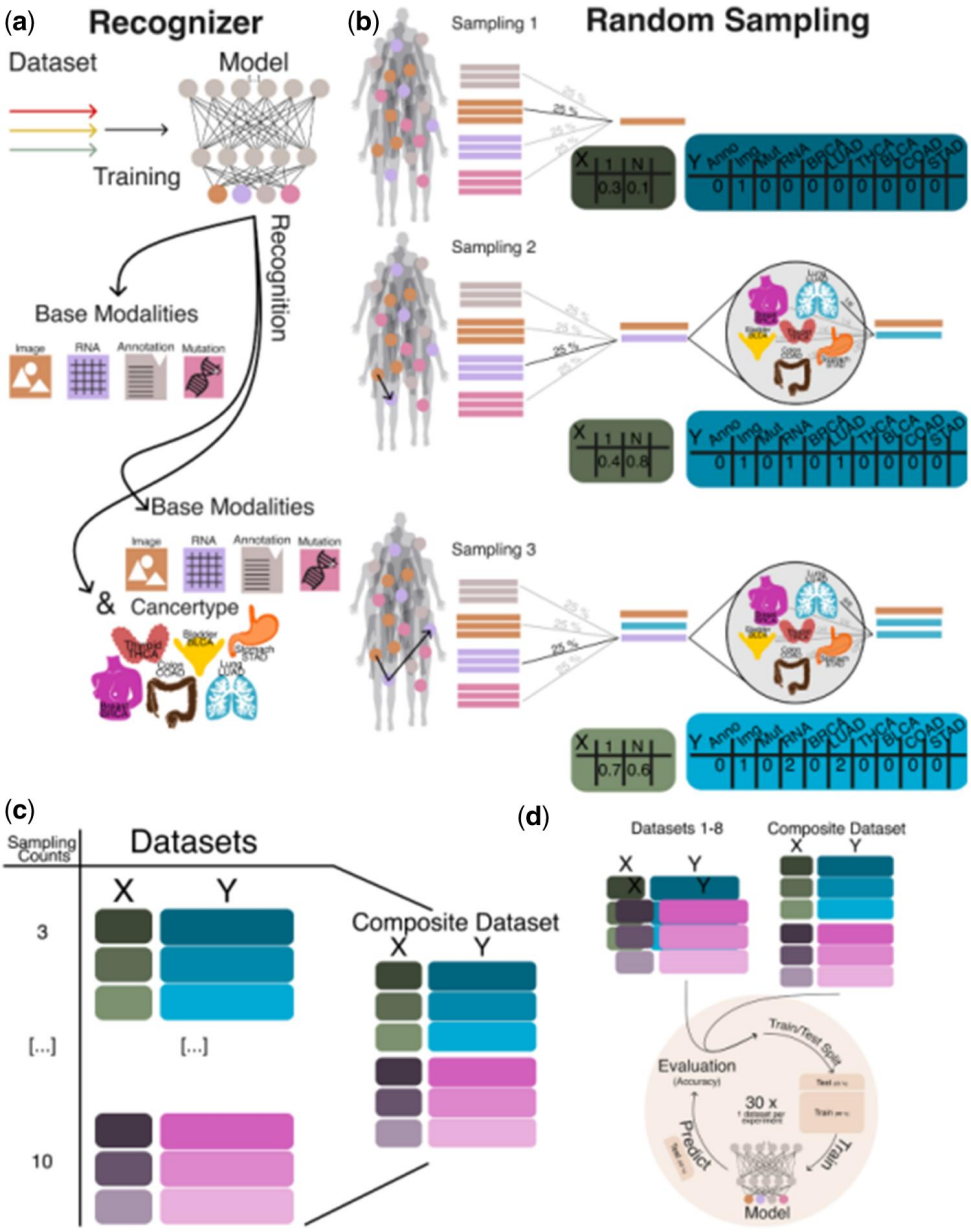
Embedding generation for the recognizer network. (a) Recognizer setup including a simple recognizer trained to identify base modalities (RNA, image, somatic mutations, and annotations), and a cancer-specific recognizer designed to additionally distinguish the cancer type from which embeddings are derived. (b) Random sampling (RS) process shown for a sample count of 3. The process begins by selecting a random embedding (X) and recording its modality and cancer type in Y. A second embedding is sampled from the same cancer type, summed with the first, and tracked. A third embedding is then added, completing the pooled vector while continuing to track contributing modalities. (c) Composite dataset generation by combining all datasets generated using sample counts ranging from 3 to 10. (d) Both isolation and composite datasets are used for training and evaluating model performance, with training and testing conducted using at least n>30 independent runs per condition.

To assess whether cancer-type structure is intrinsically preserved in the pooled embedding space, we applied GMM clustering to patient-level embeddings generated with a sample count of 3 and three sampling repeats (Fig. 2a). At this lower sampling depth, clusters showed substantial overlap (Fig. 2b), and silhouettes using cancer labels were near zero (0.0018 Euclidean, 0.025 cosine), although cancer-type signal was still detectable: adjusted rand index (ARI) was 0.066, normalized mutual information (NMI) was 0.132, and purity and class coverage were 0.38 and 0.33. Increasing the sampling depth to a sample count of 5 with five repeats markedly improved clustering behavior. The association between GMM components and cancer labels strengthened (ARI 0.24, NMI 0.35, homogeneity and completeness ∼0.35), and purity and class coverage increased to ∼0.53. Hungarian-mapped accuracy and macro-F1 reached 0.45, substantially above the random baseline, and a chi-square test confirmed a highly significant dependence between clusters and cancer type. Although cancer types did not form compact geometric clusters, higher sampling depth produced clearer separation in PCA space (Fig. 2c and d) and strong enrichment of specific cancers within individual GMM components (Figs 1–3, available as supplementary data at *Bioinformatics Advances* online). These findings show that cancer identity is robustly retained in the pooled embedding space and that unsupervised clustering quality increases substantially with sampling richness (Tables 3 and 4).

**Table 3:** Statistical evaluation of clustering using a sample count of three and three sampling repeats indicates limited cancer-type separation at low sampling depth.

Metric	Score
Silhouette Euclidean (true/GMM)	0.001830/0.0114
Silhouette Cosine (true/GMM)	0.0254/0.0581
ARI	0.065
NMI	0.131
Homogeneity	0.13
Completeness	0.13
V-Measure	0.13
Purity	0.38
Hungarian accuracy	0.30
Macro-F1	0.312
Chi^2^ *P* value	.0

**Table 4:** Statistical evaluation of clustering using a sample count of 5 and sampling repeat of 5, showing that clustering demonstrates substantial cancer-type signal retention.

Metric	Score
Silhouette Euclidean (true/GMM)	0.0109/0.025
Silhouette Cosine (true/GMM)	0.0375/0.0479
ARI	0.24
NMI	0.35
Homogeneity	0.34
Comp (completeness)	0.35
V-Measure	0.35
Purity	0.52
Hungarian accuracy	0.45
Macro-F1	0.53
Chi^2^ *P* value	.0

### 4.2 Identification of component embeddings in pooled representations

In this portion of the study, we evaluated the ability of machine learning models to accurately identify the underlying data modalities and cancer types used to generate each pooled embedding. Successful recognition of the constituent modalities and cancer type supports our hypothesis that, despite the naive combination of embeddings, sufficient structural information is preserved to enable meaningful downstream analyses. To support this hypothesis, we implemented two modeling strategies: a simple approach, where the model was tasked with distinguishing between the base modalities (e.g. RNA, image, mutations, and annotations), and a cancer-specific approach, which further required the model to identify the cancer type from which the embeddings were derived (Fig. 3a).

To generate datasets for training and evaluation of the recognition model, we created samples using between 3 and 10 random sampling steps, with each dataset comprising 15 000 data points (Fig. 3b). To prevent data leakage between training and test sets, patients were assigned exclusively to either the training or test set, ensuring that all data from a given patient were used in only one partition.

**Figure 4:**
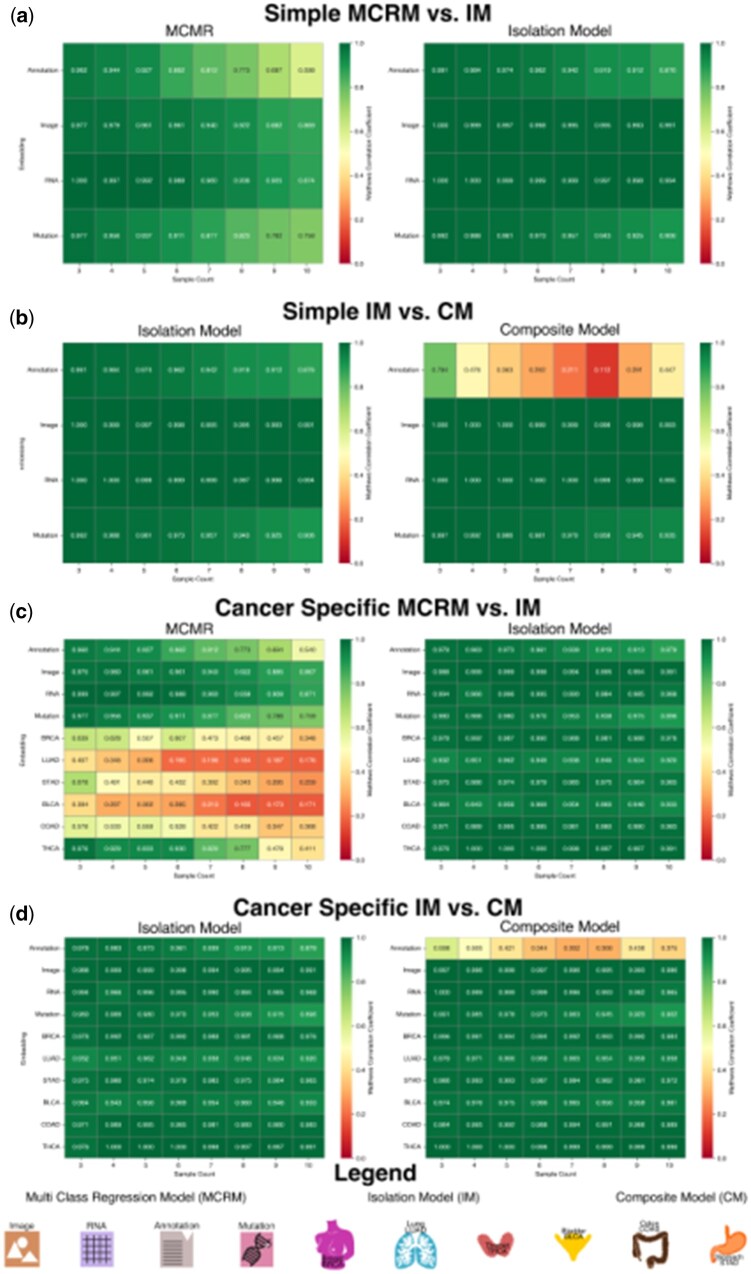
Performance of deep learning and baseline models across pooling strategies and sampling contexts. (a) Performance of the simple pooling approach using isolation models (IM) across modalities, evaluated at sample counts ranging from 3 to 10. The multi-class logistic regression model (MCRM), used as a baseline, achieves MCC values between 0.6 and 0.8, while the DL-based IM consistently outperforms it, achieving MCC values above 0.9. (b) Comparison between composite models (CM) and isolation models (IM) under the simple pooling setting. Performance remains high across most modalities for both models, except for the annotation modality, where the CM shows a marked decline in MCC with increasing sample count, dropping as low as 0.4. (c) Performance under the specific-cancer approach comparing cancer-specific IMs to the MCRM baseline. The baseline shows poor performance for several cancer types, particularly BLCA, LUAD, and STAD, with MCC values between 0.2 and 0.4. In contrast, the DL-based IMs achieve strong and consistent performance across all cancer types (MCC > 0.9). (d) Comparison between cancer-specific composite models (CM) and isolation models (IM). While most modalities maintain high performance (MCC > 0.85) across both models, the annotation modality shows poor performance in the CM across all sample counts, consistent with trends observed in panel b.

To assess model generalizability, we constructed a composite dataset by integrating all datasets containing between 3 and 10 constituents, resulting in a total of 90 000 summed embeddings (Fig. 3c). To establish a baseline, we employed a multiclass logistic regression model (MCRM) to evaluate the feasibility of recognizing the composition of pooled embeddings, aiming to determine whether the model could accurately distinguish the base modalities used to generate each pooled vector and to evaluate whether more complex models are necessary. Additionally, we developed a deep learning (DL) model to leverage a more expansive feature space for representation learning. Both models were trained and evaluated on datasets split into training and test sets, with each experiment count of n≥30 to ensure robustness (Fig. 3d). Given the dataset’s class imbalance and zero inflation, we evaluated model performance using the MCC, balanced accuracy, and F1 score. MCC was chosen as the primary metric due to its suitability for imbalanced data (Jurman *et al.* 2012, Chicco *et al.* 2021). Figure 4a illustrates model performance under the simple pooling approach, which corresponds to the first strategy described earlier. In this setting, we trained isolated models, where each model was exposed only to embeddings generated using a fixed number of randomly sampled modality vectors (referred to as sample count, e.g. 3). Sample count serves as a proxy for pooling complexity, with higher counts introducing more heterogeneity into each embedding. Both the MCRM baseline (BL) and DL models achieved high MCC, accuracy, and F1 scores at lower sample counts. However, as the number of pooled vectors increased, performance differences became more pronounced—particularly for somatic mutation and annotation embeddings. The MCC for the MCRM BL model declined below 0.8 for somatic mutations and below 0.6 for annotations, while the DL model maintained stable performance close to 0.9 across all sample counts.

Figure 4b compares two DL models: an isolation model, trained separately on embeddings generated from a fixed sample count, and a composite model, trained on a dataset comprising all sample counts. Across most modalities, both models performed similarly. However, for annotation embeddings, the composite model consistently underperformed relative to the isolation model. Specifically, the composite model’s MCC ranged between 0.2 and 0.6, while the isolation model maintained higher and more stable MCC values, demonstrating greater robustness when trained on uniformly structured inputs. We hypothesize that this discrepancy stems from both the nature of the embeddings and the interaction between embedding heterogeneity and pooling complexity. Notably, the annotation embeddings were generated using sBERT, whereas all other modality embeddings were generated using VAEs. As a result, the annotation embeddings originate from a fundamentally different and more semantically structured latent space, making them less compatible with VAE-based embeddings during pooling. This heterogeneity likely contributes to the model’s difficulty in learning consistent representations when multiple annotation embeddings are combined in a composite setting. At lower sample counts (e.g. 3), the pooling of a small number of heterogeneous embeddings may still preserve relatively coherent inter-modality patterns, enabling the model to extract useful signals despite differences in latent space. However, as the sample count increases, feature incompatibility across modalities becomes more pronounced. The model struggles to reconcile divergent feature distributions and representations not designed to be directly comparable, leading to a marked decline in performance as the sample count increases from 3 to 8. Interestingly, performance begins to recover at higher sample counts (around 9 or more), suggesting that sufficient pooling of diverse embeddings may allow the model to detect higher-order patterns across modalities. At this point, redundancy and complementary information across heterogeneous embeddings may help the model to abstract away modality-specific noise and form more robust decision boundaries. Certain cancer-related features may become detectable across multiple modalities, allowing the model to generalize effectively despite latent space mismatches.

Figure 4c presents results from the specific-cancer approach, where isolated models were trained using a fixed number of samples per cancer type (e.g. three samples per cancer). Despite this constraint, the DL model maintained stable performance across cancer types. In contrast, the MCRM BL model exhibited significant performance degradation under these conditions, with the effect particularly pronounced for cancer types such as LUAD and BLCA, where MCC dropped to 0.2 when trained on a sample count of 10.

Figure 4d mirrors the composite versus isolation comparison within the specific-cancer context, again evaluating only DL models. Consistent with the findings in Fig. 4b, annotation embeddings exhibited the most pronounced performance drop in the composite setting. The composite model failed to achieve MCC values above 0.6 for annotation embeddings, with performance falling below 0.4 for sample counts between 5 and 8. In contrast, the isolation model maintained consistently high performance, with MCC values remaining above 0.9 across all sample counts.

We attribute this pattern to the same factors observed in Fig. 4b, with an important clarification regarding the nature of the underlying embedding spaces. Annotation embeddings are generated using sBERT, which produces deterministic embeddings, each input is mapped to a fixed point in a high-dimensional semantic space without an explicit distribution. In contrast, embeddings from other modalities are derived from VAEs, which learn probabilistic latent spaces and encourage structured, continuous representations. The VAE architecture supports smooth interpolation and pooling of latent vectors, properties that are not inherently preserved in the sBERT embedding space.

As a result, when annotation embeddings are combined with VAE-based embeddings in the composite setting, especially across varying sample counts and cancer types, the lack of compatibility between these embedding spaces likely introduces feature conflicts that degrade model performance. Isolation models, by contrast, operate on more uniformly structured inputs and are thus less affected by this latent space mismatch, enabling them to maintain stable performance even when incorporating heterogeneous modality types.

**Figure 5:**
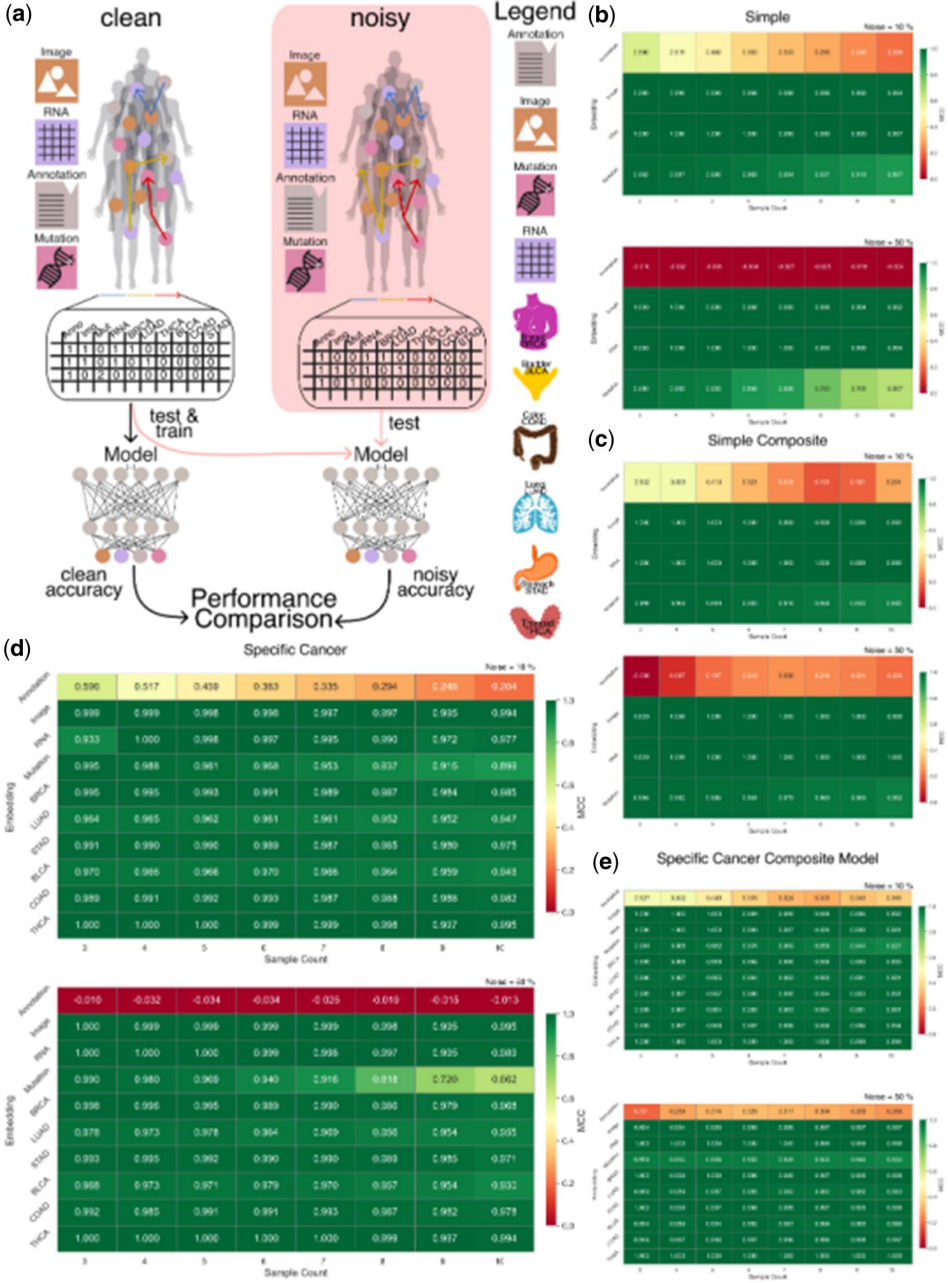
Performance of deep learning recognizer models under controlled noise perturbation. (a) Overview of the experimental setup: models were trained on clean (noise-free) pooled embeddings and evaluated on test data with controlled Gaussian noise introduced at 10% and 50% of embedding positions. Noise was applied while preserving the original sample count to assess robustness in distinguishing true modality embeddings from noise. (b) Performance of the simple pooling approach using isolation models (IM) under noisy conditions. At 10% noise, the model maintains high MCC values for RNA and image modalities, while performance for the mutation modality decreases moderately and the annotation modality shows poor performance even at low noise levels. At 50% noise, annotation performance remains consistently low, and mutation modality performance declines progressively with increasing sample count. (c) Performance of the specific-cancer approach using isolation models (IM) at 10% and 50% noise. Models demonstrate stable MCC values across most modalities at 10% noise, with the exception of annotations. At 50% noise, annotation performance further deteriorates, and mutation embeddings exhibit notable decline for sample counts greater than 6. (d) Performance of the simple pooling composite model (CM) under noisy conditions. Despite the presence of noise, the model achieves high MCC values (>0.9) for most modalities at both 10% and 50% noise. The annotation modality continues to underperform across all conditions. Notably, mutation embeddings show improved stability compared to the simple isolation model. (e) Performance of the specific-cancer composite model (CM) under 10% and 50% noise. The model maintains strong MCC performance across most modalities and cancer types, except for the annotation modality, which consistently exhibits poor performance across both noise levels.

To further evaluate model robustness, we introduced a controlled noise perturbation framework in which a defined proportion of modality embeddings within each pooled vector was systematically replaced with random Gaussian noise. This procedure preserved the total number of embeddings per vector—maintaining the original sample count, and enabled assessment of the model’s ability to differentiate true modality embeddings from noise. The DL model was trained exclusively on clean (noise-free) data and subsequently evaluated on perturbed datasets. Performance was assessed using MCC, F1 score, and balanced accuracy to quantify the impact of noise on the model’s ability to correctly infer embedding composition (Fig. 5a).

Due to the MCRM baseline model’s limited performance in previous experiments, this analysis was conducted exclusively with the DL model. We evaluated both the simple pooling approach and the specific-cancer approach, comparing model performance across increasing noise levels. Figure 5b–e presents results across noise conditions ranging from 10% to 50% embedding replacement.

Under the simple pooling approach using isolation models (Fig. 5b and c), performance remained robust at lower noise levels. Mutation embeddings, in particular, demonstrated high resilience, maintaining MCC values above 0.8 when 10% of the input was replaced with noise. However, at 50% noise, mutation performance declined to approximately 0.6 MCC, suggesting reduced but still meaningful predictive capacity. In contrast, annotation embeddings, derived from the sBERT model rather than VAE-based encoders, exhibited consistently lower MCC values, even under minimal noise, reflecting their greater sensitivity to perturbation and underlying latent space incompatibility.

In the specific-cancer setting using composite models (Fig. 5d and e), annotation embeddings continued to perform poorly across all noise levels. However, mutation embeddings showed enhanced stability compared to the isolation setting, with MCC values exceeding 0.9 even at higher noise levels. This suggests that composite training across a broader dataset may help the model generalize more effectively in the presence of noise, at least for certain modalities.

These results underscore the DL model’s resilience to input perturbations and highlight the importance of both pooling strategy and embedding origin in determining robustness. While noise degrades performance in a modality-dependent manner, the ability to retain high MCC values, particularly in the mutation modality, demonstrates the potential of DL to support reliable composition recognition even under challenging conditions.

### 4.3 Tumor of origin identification

**Figure 6:**
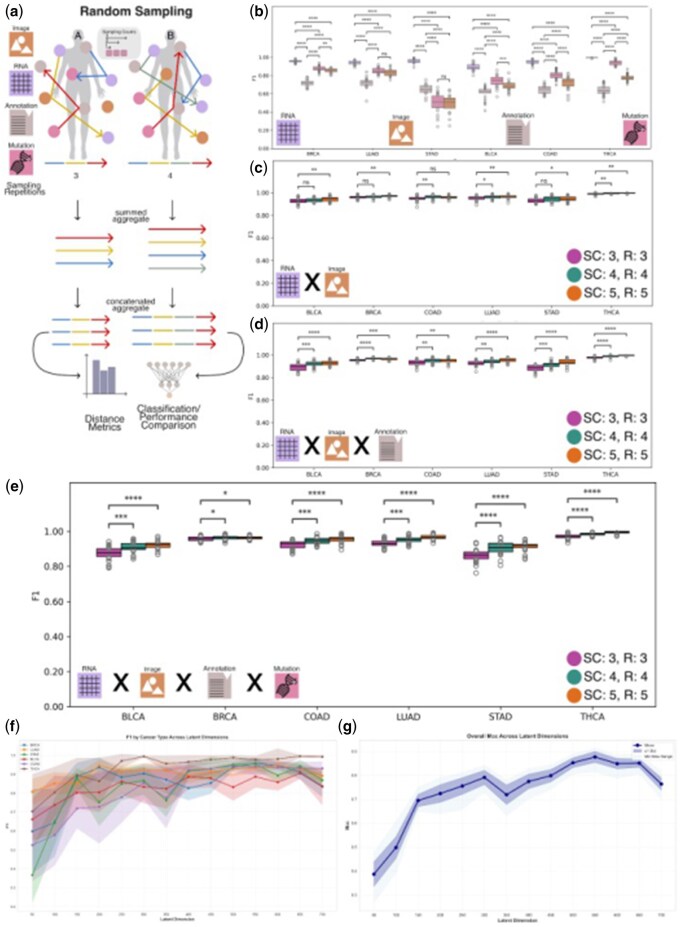
(a) Schematic illustration of patient-specific embeddings generated by pooling and concatenating vectors obtained from repeated random samplings of the graph structure. Each sampling yields an embedding vector, and concatenation produces a single patient-level representation used for downstream distance-based analyses. (b) F1 metric showing unimodal cancer type classification performance. RNA achieves the strongest performance (F1 > 0.9 across all cancer types), followed by mutations, with annotations performing the weakest. (c) F1 metric for combined RNA and image embeddings across varying sample counts (SC) and sampling repetitions (R). All cancer types show high performance (F1 > 0.9), indicating that biological signal is preserved through aggregation. (d) F1 performance of aggregated RNA, image, and annotation embeddings across SC and R combinations. Performance remains strong (F1 > 0.80) and increases with larger SC and R, further supporting signal preservation. (e) Overview of F1 scores for all modality combinations showing consistently strong performance (F1 > 0.8) across SC and R settings, with additional improvements as both parameters increase. (f) F1 values across cancer types and latent dimensions increase steadily with larger latent spaces up to approximately 550 dimensions, beyond which performance levels off, indicating a saturation of useful latent capacity. (g) MCC values (±1 SD and min-max range) similarly rise with increasing dimensionality up to around 550 dimensions, followed by a decline in performance at higher dimensions.

The classification task in this study aimed to predict specific cancer types from pooled embeddings. While the dataset remained consistent with previous experiments, this task employed the second sampling strategy, patient-level sampling, to ensure that each pooled embedding corresponded uniquely to a single patient. Each embedding integrated heterogeneous modality representations, including RNA-seq, H&E image features, clinical annotations, and somatic mutation profiles, reflecting the structure of real-world clinical datasets where multimodal information is available and is ideally combined for tasks such as cancer type prediction. To prevent data leakage, patients were assigned exclusively to either the training or test set. Pooled embeddings were then constructed by performing multiple rounds of random sampling (sample repetitions) per patient. In each round, a subset of that patient’s modality-specific embeddings was randomly selected and summed to produce a single embedding of fixed dimensionality (e.g. 768 dimensions). To capture additional intra-patient variability and enhance representational richness, this sampling process was repeated multiple times. The resulting sampled embeddings were then concatenated to form a final, patient-specific pool embedding. For example, if three sampling rounds were performed, the final embedding would have a dimensionality of 3*768=2304. This approach preserved both the diversity of intra-patient modality contributions and a standardized input structure suitable for downstream machine learning analysis (Fig. 6a).

Before evaluating classification models, we conducted an exploratory analysis using traditional vector similarity metrics, including Euclidean distance, cosine similarity, and dot product, to assess whether the pooled embeddings inherently reflect cancer-type-specific structure. This analysis was motivated by the widespread use of such metrics in vector database retrieval systems, where embedding similarity governs search and retrieval operations. By comparing intra-cancer and inter-cancer distances, we evaluated whether these metrics alone could meaningfully differentiate between cancer types. As shown in Fig. 6b and Fig. 4, available as supplementary data at *Bioinformatics Advances* online, patient embeddings from the same cancer type exhibited higher similarity to each other than to embeddings from other cancer types. These findings indicate that even in the absence of supervised learning, pooled embeddings retain informative structure relevant to cancer identity and may be applicable in retrieval-based clinical systems.

### 4.4 Tumor classification using a DL model

For DL-based classification, we used single modality embeddings to establish a baseline performance for each modality (Fig. 6b). After establishing the baseline, we then performed ablation experiments, using all available modality combinations (e.g. RNA × image, mutation × annotations) (Fig. 6c–e). These embeddings integrated information obtained from multiple random samplings of each patient’s modality-specific data, capturing both the heterogeneity and redundancy across input modalities. Model performance, evaluated using F1 score (Fig. 6b–e, Fig. 5, available as supplementary data at *Bioinformatics Advances* online), demonstrated the effectiveness of this approach in predicting cancer types from multimodal patient-level embeddings. Across all cancer types, the model consistently achieved F1 scores ≥0.8 and accuracy scores ≥0.7, when using all available modalities. The lowest performance was observed for BLCA and STAD. As shown in the confusion matrix (Fig. 6d), misclassifications frequently occurred between LUAD and BLCA, as well as between COAD and STAD, consistent with previously reported molecular overlaps (Chang *et al.* 2018).

To investigate the influence of sampling design, we evaluated how model performance varied with different sample counts and sampling repetitions. As shown in Fig. 6e (MCC) and Fig. 5, available as supplementary data at *Bioinformatics Advances* online (F1 score), increasing either the number of embeddings per sample or the number of sampling rounds consistently improved classification performance. These results suggest that pooling additional heterogeneous information from a patient, regardless of the specific modality, enhances patient-level representation and improves the model’s ability to differentiate between cancer types.

To extend this framework to additional predictive tasks, we evaluated the feasibility of using the same pooled embeddings to classify cancer subtypes (Figs 6 and 7, available as supplementary data at *Bioinformatics Advances* online) and to predict TMB (Figs 8 and 9, available as supplementary data at *Bioinformatics Advances* online). The model achieved reasonable performance across both tasks, further supporting the utility of integrating multiple unaligned modalities to construct a robust, patient-specific embedding suitable for a wide range of clinically relevant predictions.

### 4.5 Impact of latent dimensions for performance

The dimensionality of the latent space plays a central role in determining the representational capacity of embedding-based models, particularly when integrating heterogeneous biomedical modalities. In this study, the latent space dimension for the VAE was set to 768, because of predefined sBERT dimensions. To systematically evaluate the impact of latent space dimensionality on model performance and to verify that increased capacity did not artificially enhance results, we conducted a controlled experiment by varying the latent dimension from 50 to 700 in increments of 50, using the combined RNA and mutation modalities.

As shown in Fig. 6f and g and Figs 10–13, available as supplementary data at *Bioinformatics Advances* online, performance initially varied substantially across cancer types using 50 dimensions, with low F1 scores for STAD (F1<0.4) and relatively high performance for LUAD (F1>0.8). However, as the latent dimensionality increased, classification performance improved consistently across all cancers, reaching an optimal range at approximately 500–550 latent dimensions. Beyond this point, performance plateaued or exhibited slight declines, suggesting that further increases in dimensionality primarily captured redundant or noisy variation rather than meaningful biological signal. This trend indicates that the joint RNA-mutation manifold exhibits a high intrinsic dimensionality, consistent with the complexity and sparsity of these molecular datasets. Importantly, the observed plateau at 500–550 dimensions suggests that this range represents the model’s effective information capacity, large enough to encode relevant multimodal structure without overparameterization.

Previous publications have shown that as few as 82 dimensions are sufficient to encode a single phenotype from expression data (Way and Greene 2018, Chow *et al.* 2022). The effect we note is functional in these lower bound experiments, with signal recoverable at 50 dimensions. Although power is diminished at these lower dimensionalities this effect improves consistently as dimensionality increases. The capacity of a high-dimensional space to support distinct, non-interfering signal directions grows with dimensionality: the Johnson-Lindenstrauss lemma establishes that the number of quasi-orthogonal directions that can coexist in a d-dimensional space increases exponentially with d, making cross-space interference geometrically less probable as d grows Luisto (2025). By this measure, the 500–550 dimension threshold we observe empirically represents a point at which the RNA-mutation manifold is sufficiently separated from random noise. The optimal range is well below the 768 dimensions used by foundation models such as GeneFormer (Zheng and Gao 2023). Together, these results indicate that unaligned pooling benefits substantially from the high-dimensional geometry characteristic of modern foundation models, and that this method is best applied in that context. Thus, best application of this method should be targeted as foundation model–based embeddings, and their much higher dimension counts.

## 5 Discussion

Our findings demonstrate that the performance of both the recognizer and the classifier model indicates that embeddings from diverse embedding spaces can be effectively combined to describe local subgraphs without the need of a shared latent space. This approach enables the successful identification of both the composition of pooled embeddings and the cancer type, sub-type, or TMB associated with them.

Furthermore, our findings demonstrate that in a heterogeneous graph, poolings covering specific regions can serve as a secondary index, enabling the linkage of multimodal records to individual entities. Our results highlight the feasibility of using heterogeneous networks for graph neural network transformations, expanding the potential applications of these architectures. Vector databases have gained significant attention as a foundational component of retrieval-augmented generation (RAG) systems. Algorithms such as Hierarchical Navigable Small Worlds (HNSWs) (Malkov and Yashunin 2020) allow for the rapid indexing of vector-based data. This work demonstrates that despite mixing uncoordinated embedding spaces, pooled vectors still contain sufficient data to be identifiable regarding their modality composition. This will allow for indexing of complex heterogeneous data graphs, not only at the per node level, but also at the neighborhood level. Records in a complex patient information system can be rapidly compared, even if the assays available are not equivalent between each individual.

We hypothesize that the observed performance degradation in the annotation modality stems from fundamental differences in the embedding generation methods. While all other modalities were encoded using VAEs, annotation embeddings were generated using sBERT. This architectural mismatch likely contributed to the reduced performance of the annotation embeddings, particularly in recognition tasks and under composite training conditions. VAEs are designed to construct a continuous, structured latent space by enforcing a probabilistic distribution over the learned representations. This structure promotes smooth interpolation, supports meaningful pooling, and enhances compatibility across embeddings—properties that are critical for robust downstream learning. In contrast, sBERT produces deterministic embeddings optimized for semantic similarity, without enforcing continuity or geometric smoothness in the latent space. As a result, sBERT-derived embeddings may exhibit a more fragmented or irregular structure, with weaker alignment to the representations learned by VAEs. This mismatch likely becomes more problematic as the sample count increases in pooled embeddings, introducing feature conflicts that the model struggles to reconcile. Specifically, when a small number of embeddings (e.g. sample count of 3) are combined, the model may still learn relatively coherent patterns between modalities, even in the presence of latent space heterogeneity. However, as additional embeddings are pooled, the lack of alignment between the sBERT and VAE embedding spaces amplifies inconsistency, degrading performance—particularly in composite models where embeddings from a wide range of sample counts and cancer types are mixed. Interestingly, at higher sample counts (e.g. 9 or more), we observed a partial recovery in model performance. We hypothesize that this is due to the emergence of higher-order patterns and redundancies across modalities. As more diverse data are included in the pooling, the model may begin to abstract away modality-specific inconsistencies and instead learn robust decision boundaries that generalize across latent spaces. Nevertheless, the persistent underperformance of annotation embeddings, especially in composite settings, underscores the importance of selecting compatible embedding strategies when integrating unaligned modalities. These findings suggest that the structural properties of embedding spaces—particularly whether they support compositionality, smoothness, and alignment—play a critical role in determining model robustness and generalization in multimodal learning frameworks.

Our results also show that latent dimensionality plays a critical role in determining the stability and informativeness of multimodal embeddings. By systematically varying the latent space size, we observed consistent performance gains across cancer types as dimensionality increased, with models reaching an optimal range at approximately 500–550 dimensions. Beyond this point, performance plateaued or declined, suggesting that additional capacity primarily captured redundant variation rather than meaningful biological structure. These findings indicate that the joint RNA-mutation embedding space has a relatively high intrinsic dimensionality and that appropriately sized latent spaces are essential for preserving complementary signals across modalities while avoiding overparameterization.

These key aspects of heterogeneous embedding pooling help to identify how it should be situated in a larger analytical framework. First, it should be applied to the integration of large foundation models with sufficient dimensionality. Second, it should be deployed when more conventional means are not possible or practical. Again, in the context of foundation models it is often impractical or impossible to build a joint embedding using models trained against petabytes of data over hundreds of thousands of GPU hours. Third, it can quickly be deployed at low cost in cases where a large number of new joint samplings need to be generated rapidly. An example of this would be using the method to create a vector index to enable rapid search of a large healthcare data system that has already been populated by a number of proprietary or vendor embedding models already deployed on multimodal patient data. This technique can be applied post hoc on existing data, allowing multimodal sampling and indexing where it had not been previously planned.

Future work could explore alternative embedding strategies for annotations, such as fine-tuned VAEs or hybrid approaches that combine semantic and latent-space regularization, to improve consistency across modalities. A key application would be benchmarking heterogeneous data graphs, but identifying a multimodal data graph with complex relationships, structures, and well-defined prediction tasks will take considerable effort. This article has demonstrated that the basic premise of multimodal embedding pooling is viable and is able to hold information without interfering signals immediately collapsing into noise. We plan to apply this technique to encoding tumor evolution patterns, including encoded information representing tri-nucleotide patterns, structured somatic mutation timing, and subclonal mutation clustering. Other experiments could include multimodal single cell data encoding, capturing transcriptomic and methylation data across various cell states.

## Supplementary Material

vbag159_Supplementary_Data

## Data Availability

The code underlying this article is available at GitHub at (https://github.com/EllrottLab/heterogeneous-embedding-vectors).
